# Pap Smear Images Classification Using Machine Learning: A Literature Matrix

**DOI:** 10.3390/diagnostics12122900

**Published:** 2022-11-22

**Authors:** Nur Ain Alias, Wan Azani Mustafa, Mohd Aminudin Jamlos, Hiam Alquran, Hafizul Fahri Hanafi, Shahrina Ismail, Khairul Shakir Ab Rahman

**Affiliations:** 1 Faculty of Electrical Engineering & Technology, Universiti Malaysia Perlis, UniCITI Alam Campus, Sungai Chuchuh, Padang Besar 02100, Perlis, Malaysia; 2 Advanced Computing (AdvCOMP), Centre of Excellence, Universiti Malaysia Perlis (UniMAP), Pauh Putra Campus, Arau 02600, Perlis, Malaysia; 3 Faculty of Electronic Engineering & Technology, Universiti Malaysia Perlis, UniCITI Alam Campus, Sungai Chuchuh, Padang Besar 02100, Perlis, Malaysia; 4 Department of Biomedical Systems and Informatics Engineering, Yarmouk University, Irbid 21163, Jordan; 5 Department of Computing, Faculty of Art, Computing and Creative Industry, Universiti Pendidikan Sultan Idris, Tanjong Malim 35900, Perak, Malaysia; 6 Faculty of Science and Technology, Universiti Sains Islam Malaysia (USIM), Bandar Baru Nilai 71800, Negeri Sembilan, Malaysia; 7 Department of Pathology, Hospital Tuanku Fauziah, Kangar 02000, Perlis, Malaysia

**Keywords:** cervical cancer, cell classification, review, SLR

## Abstract

Cervical cancer is regularly diagnosed in women all over the world. This cancer is the seventh most frequent cancer globally and the fourth most prevalent cancer among women. Automated and higher accuracy of cervical cancer classification methods are needed for the early diagnosis of cancer. In addition, this study has proved that routine Pap smears could enhance clinical outcomes by facilitating the early diagnosis of cervical cancer. Liquid-based cytology (LBC)/Pap smears for advanced cervical screening is a highly effective precancerous cell detection technology based on cell image analysis, where cells are classed as normal or abnormal. Computer-aided systems in medical imaging have benefited greatly from extraordinary developments in artificial intelligence (AI) technology. However, resource and computational cost constraints prevent the widespread use of AI-based automation-assisted cervical cancer screening systems. Hence, this paper reviewed the related studies that have been done by previous researchers related to the automation of cervical cancer classification based on machine learning. The objective of this study is to systematically review and analyses the current research on the classification of the cervical using machine learning. The literature that has been reviewed is indexed by Scopus and Web of Science. As a result, for the published paper access until October 2022, this study assessed past approaches for cervical cell classification based on machine learning applications.

## 1. Introduction

Cervical cancer is a major disease that seriously threatens women’s health [1,2]. This cancer is also known as the second most commonly affected and killer type of cancer among women around the world [3]. It results from a chronic infection of the skin and mucosal cells in women’s vaginal regions. The fact that this cancer does not manifest any signs when it first appears is the most alarming feature of it [4]. In a report by Elakkiya et al. [5], it has been mentioned that this type of cancer is curable with early detection in the early stage. Unfortunately, the mortality rate is getting higher among women around the world that have been affected by this cancer [6,7,8]. The traditional method of manual inspection, also known as a Pap smear examination, is inaccurate due to human error that may lead to a false patient diagnosis [9,10]. The technology for automated cervical cancer screening is indeed very significant for lessening the risk of cervical cancer. However, the existing approach using machine learning has drawbacks, including poor generalisation capacity in complicated situations, as well as low efficiency, accuracy, and generalisation ability [11]. Several studies have attempted to investigate the ability of machine learning to classify cervical cancer cells for the purpose of enhancing manual screening [10,12,13]. The most often used approach for predicting characteristics from a high-dimensional collection of cancer imaging data is the random forest approach [14,15,16]. However, if a large number of decision trees are utilised, the random forest approach might become too sluggish and ineffective for real-time predictions [10]. In addition, current classification approaches, such as deep learning (DL) or hand-crafted techniques, mostly rely on single detection structures and have high processing complexity and low accuracy [17].

Cervical cytopathology image classification is an important method for diagnosing cervical cancer [18]. Moreover, previous studies have mentioned that cervical cell classification has important clinical consequences in cervical cancer screening at an early stage [19,20]. The effective classification of Pap smear cell images may be used to create automated and precise cervical cancer classification systems for early diagnosis [9]. To aid in the earlier detection and diagnosis procedure of cervical cancer, the proper screening of Pap smear images is vital. In a study by Janiesch et al. [21], DL has had a huge impact on several sectors of science in recent years. It has resulted in considerable advances in speech recognition [22,23,24] and image recognition [25,26]. Prior to the development of DL, many of these tasks were regarded as being beyond the capabilities of computers, even in science fiction literature. However, DL methods are proposed to be able to compensate for the problem through computer-aided systems for cancer cell classification [8,12,13,17,24,26,27,28,29,30]. Hence, this study’s objective is to review the current development in technologies for cervical cell classification using machine learning.

## 2. Review Method

The use of modern technology in medical research has increased as a result of its advancement [31], particularly in the fields of image processing, drug discovery, computer-aided diagnosis, and cancer research [32]. A growing number of academics have adopted DL, which is known as the most popular machine learning technique for processing medical images. The medical community sees a bright future for disease prediction through machine learning [33]. There are numerous methods for cervical cell classification, in which scientists have classified cervical cancer using these methods. In recent studies, the convolutional neural network is one of the methods used to classify cell cancer through a DL process [11,18,26,27,28,34]. Apart from that, many researchers have attempted to automate the detection of cervical cancer cells using other approaches of machine learning by applying a classifier, clustering algorithm, Random Forest, Ada Boost, MLP Algorithm, feature extraction network, DeepCyto, Support Vector Machine (SVM), and others. The findings of all the proposed methods above are able to detect cancer cells with their methodologies in classifying the characteristics of cervical cancer cells. Several methods are able to achieve high-performance evaluation values in terms of accuracy, specificity, sensitivity, precision, and f-measure. Thus, this has shown that machine learning can compensate for the traditional ways of diagnosing cancer and likely detect cancer in the early stage through a screening process. A comprehensive review based on advanced searching related to the classification of cervical cancer using machine learning is the main objective of this paper.

Comprehensive review approaches are about to become the “new normal” in organizing research reviews [35]. Advanced evaluation is one of the most significant discussions currently taking place globally. The review technique entails three major steps in choosing numerous relevant papers for this study. The identification step is the first step in writing a comprehensive literature review, which includes the search for research items that may be relevant to the predetermined research question. Next, screening is done to select the inclusion and exclusion from the total searched papers. Finally, the third stage is to determine the eligibility of the paper by reviewing the abstract to identify the relevant topic and subtopic of the screened papers. The scientific literature is then reviewed and summarised in an effort to discover, choose, and evaluate the key research that has contributed to the classification of cervical cancer cells. Last, but not least, the aim of this paper is to provide suggestions for more research in response to the issues raised in this work. In this study, a comprehensive literature review is conducted using the specific method, which is a recognised best practise. Essentially, the purpose of publication rules is to aid authors in assessing the accuracy of a review by supplying pertinent and necessary information. A comprehensive review also draws attention to the randomised investigations assessments survey, which could be a key component of systematic analysis reports for various sorts of studies. Due to their reliability, the Web of Science and Scopus databases were used to analyse the research’s methodology. This section also covers the identification, screening, eligibility, and data abstraction, as discussed in the four main subsections.

### 2.1. Preliminary Identification

The identification phase involves searching for study materials relevant to the predetermined research issue of classification of a cervical cancer cell. The keywords used are ‘classification of cervical cell’. Therefore, the first step was to detect keywords and search for similar, equivalent phrases in dictionaries, thesauri, encyclopaedias, and previous research. As a result, after determining all relevant phrases, search strings for the Web of Science and Scopus databases were created (see Table 1). Thus, during the first part of the advanced searching procedure, this study effectively obtained 3048 publications from the databases.

### 2.2. Screening

The collection of possibly relevant research items is examined for content that matches the predefined research question(s) during the screening step. Content-related criteria that are frequently used in the screening phase include the selection of research items based on the classification of cervical cancer cells using machine learning. In this step, all duplicate papers will be removed from the list of searched papers. The first stage of the screening excluded 2999 publications, while the second stage examined 49 papers based on different exclusion and inclusion criteria of this study (see Table 2). The literature (research papers) was the first criterion utilised because it is the primary source of practical recommendations. It also includes reviews, meta-synthesis, meta-analyses, books, book series, chapters, and conference proceedings that were not included in the most recent study. Furthermore, the review was confined to publications in English. It is vital to remember that the strategy only focused on the year 2022. In all, four publications were rejected based on duplication criteria.

### 2.3. Eligibility

**Figure 1:**
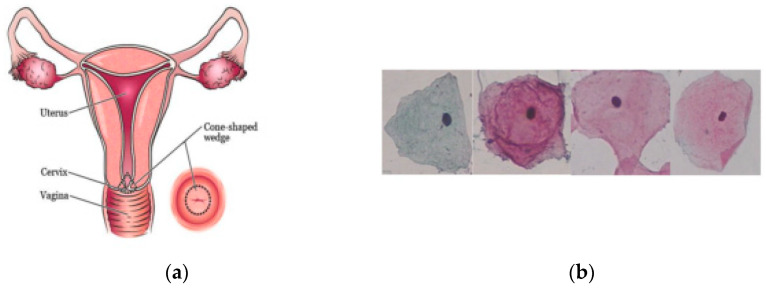
(**a**) Autonomy of women’s cervix. (**b**) Cervical cell cytology images of Herlev dataset Cervical [36].

The final review sample is generated after all inclusion and exclusion criteria have been met. A thorough disclosure of the full list of research items included in this sample is required, since readers will not know which research items exactly form the foundation for the review’s study results otherwise. The third level, termed eligibility, includes 45 articles in total. At this point, all article titles and significant content were carefully examined to ensure that the inclusion criteria were met and that the articles were relevant to the present study’s research aims. As a consequence, 18 publications were excluded, since their title and abstract were not significantly related to the study’s purpose based on empirical data. Finally, 27 papers were made available for evaluation (see Figure 1).

### 2.4. Data Abstraction and Analysis

**Figure 2:**
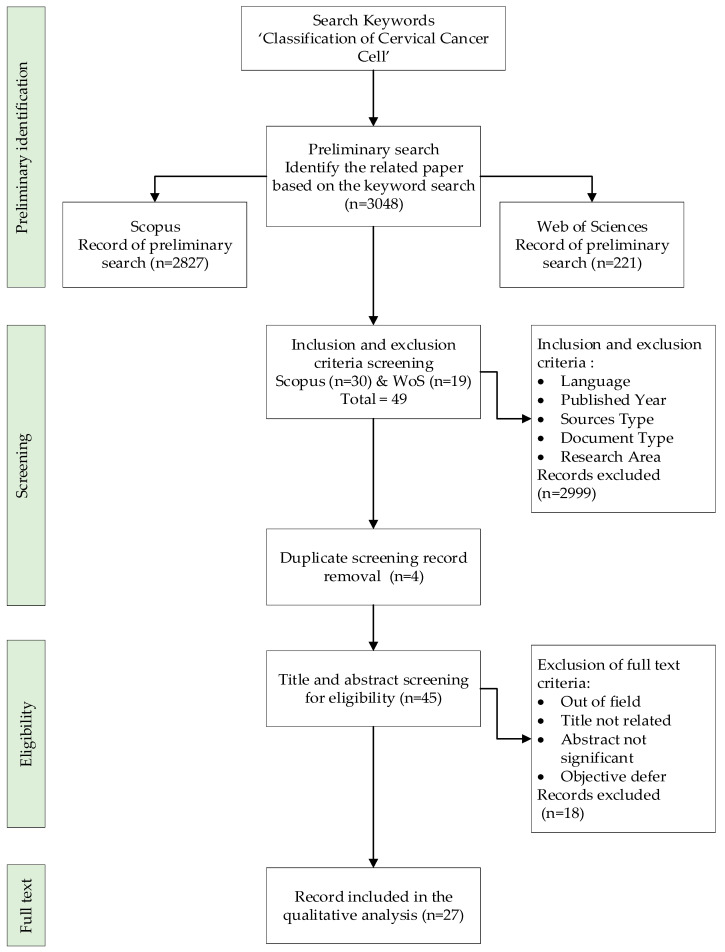
Flow diagram of the proposed advanced search study.

An integrative analysis was used as one of the assessment strategies in this study to examine and synthesise a variety of research designs (quantitative, qualitative, and mixed methods). The goal of the competent study was to identify relevant topics and subtopics. The stage of data collection was the first step in the development of the theme. Figure 2 shows how the authors meticulously analysed a compilation of 27 publications for assertions or material relevant to the topics of the current study. The authors then evaluated the current significant studies related to cervical cancer cell classification. The methodology used in all studies, as well as the research results, are being investigated. Next, the author collaborated with other co-authors to develop themes based on the evidence in this study’s context. A log was kept throughout the data analysis process to record any analyses, viewpoints, riddles, or other thoughts relevant to the data interpretation. Finally, the authors compared the results to see if there were any inconsistencies in the theme design process. It is worth noting that, if there are any disagreements between the concepts, the authors discuss them amongst themselves. The produced themes were eventually tweaked to ensure consistency. The analysis selection was carried out by two experts, one in public health (Khairul Shakir Ab Rahman—expert medical doctor in pathology) and the other in biomedical science (Wan Azani Mustafa—expert in biomedical computing), to determine the validity of the problems. The expert review phase ensures the clarity, importance, and suitability of each subtheme by establishing the domain validity.

## 3. Results and Findings

The cancer disease’s significance has increased, as public health worries about the region’s development and success. Microscopic image-based analysis has been extensively used in pathological research and disease diagnosis. However, the misauthentication of cell lines due to pathologists’ errors has been identified as a severe issue. Therefore, a comparative evaluation of the proposed model was conducted to illustrate the utility of feature selection and class imbalance based on the classifier’s accuracy, sensitivity, precision, F-measure, and specificity. The goals of this study were to improve the efficiency and accuracy of an early cervical cancer clinical diagnosis and evaluate the application of a cell classification algorithm in conjunction with multispectral imaging in cervical cancer screening. Therefore, researchers have come out with various approaches to overcome the shortcomings of the previously proposed approach for classification. The convolutional neural network is one of the famous methods for the classification of infected cells. In this study, a total of 27 articles were extracted and analysed based on the advanced searching.

### Classification of Cells Based on Machine Learning Approach

There are numerous approaches that have been done by previous researchers in the area of cell classification. The methodology and results of the approaches are summarised to make it easier to compare the findings of the studies. Table 3 illustrates the summary of the prevailing works related to the classification of cervical cancer cells using machine learning.

**Figure 3:**
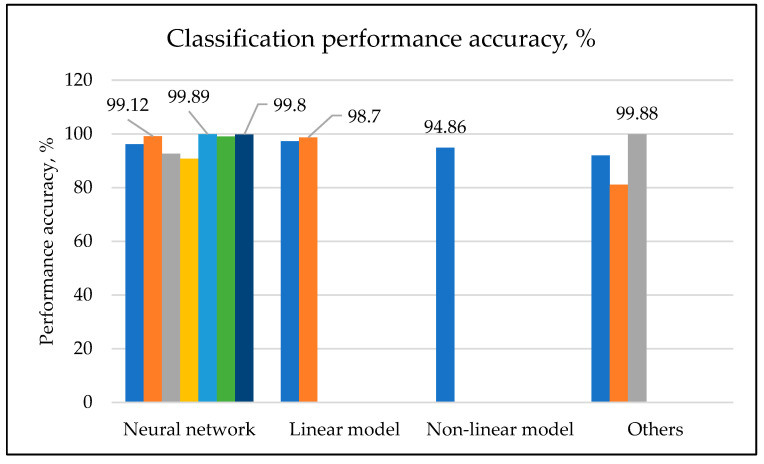
Graph of classification performance accuracy.

Among the plausible explanations for these findings is that several previous attempts have been made in the area of cervical cell classification. Convolutional neural network (CNN) is currently one of the best approaches for the classification process. Several techniques have been studied by previous researchers related to CNN, such as CNN-based long short-term memory classifier, region-based classifier, lightweight, ResNet-50, and others. For example, Chitra et al. [11] introduced a technique of classification using the Sooty Tern Optimization (STO) algorithm with a CNN-based long short-term memory classifier (CNN-LSTM) and achieved better performance results compared to other literature reviews. The results achieved in the study have shown that the accuracy is 99.80%, specificity is 99%, sensitivity is 98.83%, and F-score is 97.8. Their findings show an improvement of 28.5% better than Random Forest and 19.46% better than the ensemble classifier. The findings are consistent with the findings of the past study by Li et al. [3], which also achieved almost similar values of accuracy, specificity, sensitivity, and F-measure as the previous study by Chitra et al. [11]. The method applied was a pulse convolutional neural network (PCNN) that integrates a global context information and attention mechanism with an improved ResNet-50 backbone network for feature extraction. Other than that, in a study by Liu et al. [44], a conclusion was made that DL models are robust to changes in the aspect ratio of cervical cells in cervical cytopathological images. The above findings contradict the study by Elakkiya et al. [5], which proposed a method of identification and classification of cervical premalignant and malignant diseases based on deep characteristics without the necessity for initial classification and segmentation. The findings of the literature have come out with four different methods of classification used by previous studies, which are neural network-based classification, linear model classification, nonlinear classification model, and others. It is apparent from Figure 3 that the most popular approach in classification of cervical cells using machine learning is the neural network. The findings also provide evidence that the classification method based on the neural network have resulted in a higher accuracy level when compared to the other approaches. As illustrated in Figure 3, the neural network approach was able to achieve higher accuracy, which was more than 99%, and proved to be able to detect the class of the cell being tested in the different datasets.

## 4. Discussion and Conclusions

Cervical cancer is the second most common female cancer worldwide. It is vital to detect it earlier with low-cost, high-accuracy automated screening technologies, especially in countries with limited medical resources. Following breast cancer, this malignancy is the second biggest cause of death among women in developing countries. Utilising automatic identification, cervical intraepithelial neoplasia (CIN) can be effectively avoided. The only way to avoid morbidity is to detect the problem as soon as possible. Since the traditional Pap smear test assesses the abnormalities of the cell by hand, the clinical test used to detect cervical cancer is more prone to false-negative and false-positive results. Detecting and classifying Pap smear cell images is significantly complex in cervical cancer screening. Patients gain from earlier medical therapy when cancer is detected, diagnosed, and classified early. This study’s goal is to review the DL techniques to automate cancer diagnosis and classification in order to ensure that patient’s health conditions improve over time. The findings of a comprehensive comparison investigation revealed that the MFFOA-DL3 model outperformed other recent approaches. The suggested method identifies and classifies cervical premalignant and malignant illnesses based on deep characteristics without the need for initial classification and segmentation. The Boruta analysis shows a better performance approach compared to the existing techniques available. DeepCyto is a powerful tool for precise feature extraction and Pap smear image classification. The suggested novel screening methodology of auxiliary classification for cervical cells based on a multi-domain hybrid DL framework is promising for early cervical cancer detection, with multi-domain and hybrid characteristics proving realistic in clinical practise. All machine learning architectures gave outstanding nuclei segmentation in cervical cancer cells but did not solve the overlapping nuclei and Z-stack segmentation problems. Besides that, when it comes to coping with the complexities of large-scale data and identifying prognostic patterns, machine learning has been demonstrated to outperform traditional statistical models. It has a lot of clinical potential for enhancing cervical cancer treatment. However, the limitations of prediction studies and models, such as simplification, insufficient information, overfitting, and lack of interpretability, indicate that additional efforts are required to improve the accuracy, reliability, and practicality of clinical outcome predictions. This review paper hopes to gain significance for the better design and methodology of cervical cancer classification, with the objective of aiding the diagnosis process of this cancer detection. The proposed system architecture simplifies the detection and classification of cervical cells in Pap smear images, resulting in the early detection of cervical cancer and possibly an increase in women’s survival rates.

## Figures and Tables

**Table 1:** The search strings.

Scopus	Web of Science
TITLE-ABS-KEY (classification AND cervical AND cancer AND cell) AND (LIMIT-TO (SRCTYPE, “j”)) AND (LIMIT-TO (PUBSTAGE, “final”)) AND (LIMIT-TO (DOCTYPE, “ar”)) AND (EXCLUDE (SUBJAREA, “MEDI”)) AND (LIMIT-TO (PUBYEAR, 2022)) AND (LIMIT-TO (LANGUAGE, “English”)) Date of access: October 2022	Classification of cervical cancer cell (Abstract) or classification of cervical cancer cell (Title) or classification of cervical cancer cell (Author Key-words) and 2022 (Publication Years) and Article (Document Types) and English (Languages) and Oncology or Engineering Biomedical or Engineering Electrical Electronic (Web of Science Categories). Date of access: October 2022

**Table 2:** The selection criterion for searching.

Criterion	Inclusion	Exclusion
Language	English	Non-English
Published Year	2022	<2022
Sources type	Journal (only research articles)	Conference proceeding
Document Type	Article	Letter, Review, Conference, Note
Research Area	Computer Science and Engineering	Besides Computer Science and Engineering

**Table 3:** Summary of the prevailing works.

Title	Class and Database	Methodology	Results
CVM-Cervix: A hybrid cervical Pap smear image classification framework using CNN, visual transformer, and multilayer perceptron [18]	Database: CRIC dataset, SIPaKMeD dataset and combination of CRIC and SIPaKMeD Class: CRIC-6 class, SIPaKMeD 5 Class	Framework CVM-Cervix based on deep learning. Type: Machine learning—Neural network	Effective and potential of the proposed CVM-Cervix proved.
A Comparative Analysis of Deep Learning Models for Automated Cross-Preparation Diagnosis of Multi-Cell Liquid Pap Smear Images [37]	Database: Thin Prep Pap dataset Class: 4 Bethesda class	An ensemble novel convolutional neural network (CNN) and a CNN with autoencoder (AE). Type: Machine learning—Neural network	All models’ accuracy >905. The individual transfer model had high variability in performance, while CNN and AE CNN did not. ResNet101 accuracy is 92.65%.
Multi-class nucleus detection and classification using a deep convolutional neural network with enhanced high dimensional dissimilarity translation model on cervical cells [38]	Database: Herlev dataset, SIPaKMeD dataset, CRIC dataset Class: Herlev-7 class, SIPaKMeD-5 Class, CRIC-6 class	Segmentation—hybrid system that incorporates two binary image patches obtained by a 19-layered convolutional neural network (ConvNet) model with an enhanced deep high dimensional dissimilarity translation (HDDT). A Pre trained Resnet-50 model. T-distribution stochastic neighbour embedding (t-SNE) for down-sampled. Classification using a multi-class weighted kernel extreme learning machine (WKELM) classifier via a sparse multicanonical correlation (SMCCA) method. Type: Machine learning—Neural network	Accuracy 99.12% Specificity 99.45% Sensitivity 99.25% Execution time 99.6248 s The proposed model is more effective compared to existing approaches.
Hybrid Loss-Constrained Lightweight Convolutional Neural Networks for Cervical Cell Classification [39]	Database: Herlev dataset Class: Herlev-7 class	Classification using hybrid loss function with label smoothing to improve the distinguishing power of lightweight convolutional neural networks (CNN) Type: Machine learning—Neural network	ShufflenetV2 results:- Accuracy 96.18% Precision 96.30% Recall 96.23% Specificity 99.08% GhostnetV2 results:- Accuracy 96.39% Precision 96.42% Recall 96.39% Specificity 99.09%
Detection of cervical cells based on improved SSD network [20]	Database: Herlev dataset Class: Herlev-7 class	Integration of Single Shot MultiBox Detector with the positive and negative features to address the problem of insufficient sensitivity for small objects. Type: Machine learning—Neural network	Accuracy 90.80% Mean average precision (mAP) is 81.53%, which is 7.54% and 4.92% higher than YOLO and classical SSD.
Detection of cervical cancer cells in a complex situation based on improved YOLOv3 network [1]	Database: Herlev dataset Class: Herlev-7 class	Detection using the YOLO algorithm. Feature extraction generalization by adding the dense block and S3Pool algorithm on the basis of the feature extraction network DarkNet-53. Clustering algorithm of improved algorithm k-means++. Type: Machine learning—Neural network	Mean average precision (mAP) is 78.87%, which is 7.54% and 4.92% higher than YOLO (You Only Look Once) and classical SSD
Pap smear-based cervical cancer detection using residual neural networks deep learning architecture [27]	Database: Mendeley LBC SIPaKMeD dataset Class: Mendeley LBC SIPaKMeD—4 class	Data augmentation module of DTWCT module and convolutional neural networks (CNN). Classification using ResNet 18, which defines four classes of sources for Pap smear cell images. Type: Machine learning—Neural network	Average Pap smear detection index (PDI) is 99%.
Cervical cell multi-classification algorithm using global context information and attention mechanism [3]	Database: SIPaKMeD dataset Class: SIPaKMeD—5 class	Convolutional neural network (L-PCNN) that integrates global context information and attention mechanism. Improved ResNet-50 backbone network for feature extraction. Type: Machine learning—Neural network	Accuracy 98.89%. Sensitivity 99.9%. Specificity 99.8%. F-measure 99.89%.
DeepCyto: a hybrid framework for cervical cancer classification using deep feature fusion of cytology images [40]	Database: Herlev dataset, SIPaKMeD dataset, LBC dataset Class: Herlev-7 class, SIPaKMeD-5 class, LBC-4 class	Novel classification using DeepCyto. Principal component analysis and machine learning ensemble for classification of Pap smear images. Artificial neural network with feature fusion vectors as an input for classification. Type: Machine learning—Neural network	DeepCyto is a powerful tool for precise feature extraction and Pap smear image classification.
Classification of Cervical Cytology Overlapping Cell Images with Transfer Learning Architectures [29]	Database: Cervix93 cervical cytology image Class: 3 class	Transfer learning using deep learning convolutional neural network. Cutting edge pretrained networks: AlexNet, ImageNet, and Places 365. Type: Machine learning—Neural network	Accuracy 99.03%. Kappa coefficient showing perfect agreement. AlexNet proved a successful assistive tool for cervical cancer detection.
Optimal deep convolution neural network for cervical cancer diagnosis model [8]	Database: Herlev dataset Class: Herlev-7 class	Detection using intelligent deep convolutional neural network. Classification (IDCNN-CDC) model using biomedical Pap smear images. Noise removal using Gaussian Filter. Segmentation using the Tsallis entropy technique with the dragonfly optimization. Deep learned feature using SqueezeNet. Classification using weighted extreme learning machine (ELM). Type: Machine learning—Neural network	Higher performance of the proposed technique in terms of sensitivity, specificity, accuracy, and F-Score.
Modified metaheuristics with stacked sparse denoising autoencoder model for cervical cancer classification [9].	Database: Herlev dataset Class: Herlev-7 class	Novel Modified Firefly Optimization Algorithm with Deep Learning-enabled cervical cancer classification (MFFOA-DL3) model. Noise removal using Bilateral Filtering (BF)-based. Segmentation technique of Kapur’s entropy-based image to define affected area. Generate feature vectors using EfficientNet. Classification of the cell using MFFOA with Stacked Sparse Denoising Autoencoder (SSDA) model. Type: Machine learning—Neural network	The findings of a comprehensive comparison investigation revealed that the MFFOA-DL3 model outperformed other recent approaches.
Imaging based cervical cancer diagnostics using small object detection—generative adversarial networks [5]	Database: Herlev dataset, Colposcopy images, Clinical references Class: not applicable	An effective hybrid deep learning technique using Small-Object Detection-Generative Adversarial Networks (SOD-GAN) with Fine-tuned Stacked Autoencoder (F-SAE). Generation and discrimination of the cervical cell using Region-based Convolutional Neural Network (RCNN). Type: Machine learning—Neural network	The proposed method identifies and classifies cervical premalignant and malignant diseases based on deep characteristics without the necessity for initial classification and segmentation.
Cervical cancer diagnosis based on modified uniform local ternary patterns and feed forward multilayer network optimized by genetic algorithm [41]	Database: Herlev dataset Class: Herlev-7 class	Segmentation of the image using a thresholding approach. Feature extraction by applying a texture descriptor titled modified uniform local ternary patterns (MULTP). Classification of the cell using an optimized multilayer feed-forward neural network. Type: Machine learning—Neural network	MULTP, the proposed texture descriptor, is a generic operator that may be used to characterise texture features of images in numerous computer vision issues. In addition, the suggested optimization approach may be utilised to increase performance in deep networks.
Early cervical cancer diagnosis using Sooty tern-optimized CNN-LSTM classifier [11]	Database: Herlev dataset Class: Herlev-7 class	Augmentation process of image enhancement, image flipping, and image rotating to reduce the number of parameters necessary. Segmentation of the cancer-affected regions with the help of kernel weighted fuzzy local information c-means clustering (KWFLICM) model. Classification using the Sooty Tern Optimization (STO) algorithm with CNN-based long short-term memory classifier (CNN-LSTM). Type: Machine learning—Neural network	Accuracy 99.80%. Specificity 99%. Sensitivity 98.83%. F-Score 97.8. Improvement of 28.5% better than Random Forest and 19.46% better than ensemble classifier.
Hybrid Model for Detection of Cervical Cancer Using Causal Analysis and Machine Learning Techniques [10]	Database: Private Class: not applicable	Boruta analysis and SVM method for an efficient feature selection and prediction of the model for the cervical cell dataset. Type: Machine learning—Linear model	Boruta analysis shows a better performance approach compared to the existing techniques available.
Cervical Cancer Classification Using Combined Machine Learning and Deep Learning Approach [42]	Database: Herlev dataset Class: Herlev-2 class	Feature extraction using ResNet-101. Classification using Support vector Machine (SVM). Type: Machine learning—Linear model	Accuracy 97.30%.
Auxiliary classification of cervical cells based on the multi-domain hybrid deep learning framework [17]	Database: Herlev dataset, SIPaKMeD dataset, BJTU dataset Class: Herlev-2&7 class, SIPaKMeD-5 Class, BJTU-7 class	Deep features extraction using deep Convolutional Neural Network of pretrained Visual Geometry Group-19 (VGG-19). Hand-crafted images undergo the process of feature selection, clustering, and dimensionality reduction. Classification using a Support Vector Machine (SVM) classifier. Type: Machine learning-Linear model	Accuracy 98.70%. Sensitivity 98.20%. Specificity 98.90%. The suggested novel screening methodology is promising for early cervical cancer detection, with multi-domain and hybrid characteristics proving realistic in clinical practise.
An Evaluation of Computational Learning-based Methods for the Segmentation of Nuclei in Cervical Cancer Cells from Microscopic Images [43]	Database: Z-Stack cellular microscopy proliferation images provided by the HCS Pharma Class: not applicable	Machine learning architecture of Random Forest, Ada Boost, and MLP algorithm. Type: Machine learning—Nonlinear model	All machine learning architectures gave outstanding nuclei segmentation in cervical cancer cells but did not solve the overlapping nuclei and Z-stack segmentation problems.
Prognosis of Cervical Cancer Disease by Applying Machine Learning Techniques [4]	Database: Dataset of 858 cervical cancer patients with 36 risk factors and one outcome variable Class: not applicable	Analysis of the different supervised machine learning techniques. The classification algorithm used Artificial Neural Network, Bayesian Network, SVM, Random Tree, Logistic Tree and XG-Boost Tree. Selection algorithm for feature selection: relief rank, wrapper method, and LASSO regression. Type: Machine learning—Nonlinear model	Maximum accuracy achieved using XG-Boost with complete features 94.94%. This approach offers much potential for clinical use and cervical cancer cell detection.
Is the aspect ratio of cells important in deep learning? A robust comparison of deep learning methods for multi-scale cytopathology cell image classification: From convolutional neural networks to visual transformers [44]	Database-SIPaKMeD dataset Class: not applicable	Twenty-two deep learning models were used to classify the cervical cancer cells into two categories of standard and scaled datasets. Type: Machine learning—Nonlinear model	Deep learning models are robust to changes in the aspect ratio of cervical cells in cervical cytopathological images.
A Fast Hybrid Classification Algorithm with Feature Reduction for Medical Images [45]	Database: Herlev dataset Class: Herlev-7 class	A novel fast hybrid fuzzy classification algorithm with feature reduction for medical images. Integration of quantum-based grasshopper computing algorithm (QGH) with a fuzzy clustering technique for feature extraction. The second integration of the fusion technique utilises QGH with the fuzzy c-means algorithm to determine the best features. Type: Machine learning—Nonlinear model	Established the importance of the feature selection on the accuracy of the proposed classifier
Cervical Cancer Classification from Pap Smear Images Using Modified Fuzzy C Means, PCA, and KNN [7]	Database: Herlev dataset Class: Herlev-7 class	Geometrical and feature extraction using a novel approach of modified fuzzy c-means. Augmentation of the images using Principal Component Analysis (PCA) to maintain the uncorrelated features and thus reduce the algorithm processing time. Classification of the Pap smear image into normal and abnormal cells using K Nearest Neighbour (KNN). Type: Machine learning—Nonlinear model	Minimum accuracy 94.15%. Maximum accuracy 96.28%. Average accuracy 94.86%. Sensitivity 97.96%. Specificity 83.65%. F1-Score 96.87%. Precision 96.31%.
A Semi-supervised Deep Learning Method for Cervical Cell Classification [28]	Database: Herlev dataset, SIPaKMeD dataset Class: Herlev-7 class, SIPaKMeD-5 Class	A novel manual features and voting mechanism to achieve data expansion in semi-supervised learning. Clarity function to filter out higher-quality images, annotating a small amount of the high-quality images, and voting mechanism for balancing and training data. Type: Machine learning—Classifier	Accuracy 91.94%.
Ensemble feature extraction model with optimal kernelized clustering algorithm for identifying cancer from cervical histopathology images [46]	Database: 962 histopathological cervical images. Class: Not applicable	Provide improvement in the efficiency of the clustering network algorithm of Optimal Kernelized Fuzzy C-Means (OKFCM). Accurate histopathological image ensemble-based feature extraction model. Type: Machine learning—Clustering	Higher value of performance achieved. Precision Specificity Recall AUC Accuracy FPR FNR
Cervical cancer diagnosis based on cytology Pap smear image classification using fractional coefficient and machine learning classifiers [36]	Database: SIPaKMeD dataset Class: SIPaKMeD—5 class	Feature extraction using the discrete coefficient transform (DCT) and Haar transform. Classification using seven different machine learning algorithms for normal and abnormal Pap smear images. Optimization of feature extraction using fractional coefficient to form the five different sizes of feature vectors.	Accuracy 81.11%
Improving cervical cancer classification with imbalanced datasets combining taming transformers with T2T-ViT [19]	Database: Liquid-based cytology, Herlev dataset, SIPaKMeD dataset, Class: Liquid-based cytology—4 class, Herlev dataset—7 class, SIPaKMeD dataset—5 class	Taming transformers (CCG-taming transformers). Improve the encoder structure by introducing SE-block and MultiRes-block. Layer Normalization to standardize the data. SMOTE-Tomek Links to balance the source data set and the number of samples and weights. Classification using Tokens-to-Token Vision Transformers (T2T-ViT) combing transfer learning.	Accuracy:- 4-class liquid-based cytology Pap smear dataset 98.79%. 5-Class SIPAKMeD 99.58%. 7-Class Herlev 99.88%. Inception score (IS) 3.75. Frechet inception distance (FID) 0.71. Recall 0.32. Precision 0.65. Novel approach that applies the transformer to the generation and recognition of cervical cancer cell images.

## Data Availability

Not applicable.
